# The Interaction between Cyclin B1 and Cytomegalovirus Protein Kinase pUL97 is Determined by an Active Kinase Domain

**DOI:** 10.3390/v7082834

**Published:** 2015-08-11

**Authors:** Mirjam Steingruber, Eileen Socher, Corina Hutterer, Rike Webel, Tim Bergbrede, Tihana Lenac, Heinrich Sticht, Manfred Marschall

**Affiliations:** 1 Institute for Clinical and Molecular Virology, Friedrich-Alexander-University Erlangen-Nürnberg (FAU), 91054 Erlangen, Germany; E-Mails: mirjam.steingruber@viro.med.uni-erlangen.de (M.S.); corina.hutterer@viro.med.uni-erlangen.de (C.H.); rike.webel@viro.med.uni-erlangen.de (R.W.); 2 Division of Bioinformatics, Institute of Biochemistry, Friedrich-Alexander-University Erlangen-Nürnberg (FAU), 91054 Erlangen, Germany; E-Mails: eileen.socher@fau.de (E.S.); heinrich.sticht@fau.de (H.S.); 3 Lead Discovery Center GmbH, 44227 Dortmund, Germany; E-Mail: bergbrede@lead-discovery.de; 4 Department of Histology and Embryology, Faculty of Medicine, University of Rijeka, 51000 Rijeka, Croatia; E-Mail: tihana.lenac@medri.uniri.hr

**Keywords:** human cytomegalovirus, protein kinase pUL97, CDK ortholog, cyclin B, protein-protein interaction, kinase activity, mode of kinase-cyclin interaction, active conformation

## Abstract

Replication of human cytomegalovirus (HCMV) is characterized by a tight virus-host cell interaction. Cyclin-dependent protein kinases (CDKs) are functionally integrated into viral gene expression and protein modification. The HCMV-encoded protein kinase pUL97 acts as a CDK ortholog showing structural and functional similarities. Recently, we reported an interaction between pUL97 kinase with a subset of host cyclins, in particular with cyclin T1. Here, we describe an interaction of pUL97 at an even higher affinity with cyclin B1. As a striking feature, the interaction between pUL97 and cyclin B1 proved to be strictly dependent on pUL97 activity, as interaction could be abrogated by treatment with pUL97 inhibitors or by inserting mutations into the conserved kinase domain or the nonconserved C-terminus of pUL97, both producing loss of activity. Thus, we postulate that the mechanism of pUL97-cyclin B1 interaction is determined by an active pUL97 kinase domain.

## 1. Introduction

The human cytomegalovirus (HCMV), also referred to as human herpesvirus 5 (HHV-5), belongs to the *β-Herpesvirinae* subfamily. HCMV is a ubiquitous human pathogen, which causes severe systemic diseases in immunosuppressed patients and neonates. Due to a high seroprevalence (60%–90%), HCMV is the leading infectious cause of birth defects in developed countries [1]. For the treatment of HCMV infection, all currently approved antiviral drugs, such as ganciclovir, valganciclovir, cidofovir, and foscarnet inhibit viral DNA replication by targeting the viral DNA polymerase pUL54 [2]. However, side effects based on cytotoxicity and the induction of drug-resistant viral mutants, particularly upon long-term treatment, illustrate the need for novel antiviral compounds. Protein kinases are putative targets of new herpesviral drugs due to their important role in the regulation of HCMV replication [3,4,5,6,7,8]. Current clinical trials are investigating cyclin-dependent kinase (CDK) inhibitors, such as roscovitine, an inhibitor of CDK1, -2, -5, -7, and -9, that decreases viral DNA synthesis, production of late proteins and infectious virus particles [4]. Moreover, we previously demonstrated that the selective CDK9 and CDK7 inhibitors R22 and LDC4297 exert strong anticytomegaloviral activity in cell culture models [9]. CDKs are cyclin-dependent serine-/threonine-specific protein kinases, the activity of which is largely determined by cyclin binding. In addition to their major role in the regulation of cell cycle progression, specific types of CDKs and cyclins are also involved in transcription, splicing, epigenetic regulation, neuronal functions, stem cell regeneration, spermatogenesis, and differentiation [10]. In HCMV-infected cells, specific subsets of CDK-cyclin complexes are downregulated/suppressed (CDK4-cyclin D, CDK6-cyclin D, CDK2-cyclin A) or upregulated/activated (CDK1-cyclin B, CDK2-cyclin E), eventually resulting in an early S phase arrest termed pseudomitosis [11]. This dysregulation of the cell cycle creates an environment favorable for viral replication. Along with CDK1 and -2, CDK7 and -9 are required for efficient HCMV replication and were found upregulated in HCMV-infected cells [3,12,13,14,15].

In addition to those indirect effects on cell cycle regulation, the viral protein kinase pUL97 directly cross-talks with CDKs as it mimics CDKs in phosphorylating partly-identical substrates and apparently possesses similarities in protein structure and functionality. Based on sequence analysis and a 3D model of pUL97, the viral kinase shows structural similarity to CDK2 in the catalytic center and in functionally important residues of the ATP binding site [16]. Functional similarity was demonstrated by several experimental settings, e.g., the recombinant expression of pUL97 in a yeast complementation assay, in which pUL97 was able to rescue the proliferation of a *Saccharomyces cerevisiae* mutant lacking CDK activity [17]. In line with this finding, we and others reported that specific substrates can be dually phosphorylated by CDKs and pUL97, such as the viral mRNA transporter pUL69, nuclear lamins A/C, RNA polymerase II, EF-1δ [16,18,19,20,21,22,23,24,25,26,27] and, particularly important for virus-host interaction, the human retinoblastoma protein (Rb) [17,26]. Remarkably, CDKs and pUL97 phosphorylate Rb at identical residues [17,26]. Moreover, partially overlapping functions between CDKs and pUL97 were also postulated in light of the finding that the HCMV-inhibitory effect of the pUL97 inhibitor maribavir (MBV) was increased when CDK activity was simultaneously suppressed [11]. Although pUL97 is not strictly essential for HCMV replication, the deletion of ORF UL97 or the pharmacological inhibition of pUL97 leads to a drastic reduction in the efficiency of viral replication [28,29]. Its kinase domain includes subdomains (SD) I-XI, which are conserved (aa 337–651) within herpesviral and cellular protein kinases. Notably, the second SD contains an invariant lysine residue (K355), the replacement mutation of which resulted in a complete loss of kinase activity [16,30,31]. As published recently by our group, alternative initiation of translation at codons M1, M74, and M175 results in the expression of three pUL97 isoforms, with partly individual properties in terms of regulation of viral replication and MBV susceptibility [32,33,34]. The formation of pUL97 dimers and oligomers is based on a self-interaction domain, located within amino acids 231-280, that supports strong autophosphorylation mainly occurring at N-terminal residues [35,36,37]. Nuclear translocation of pUL97 is conferred via two classical bipartite nuclear localization sequences (NLS1 and NLS2, amino acids 6-35 and 190-213, respectively) that mediate importin-α binding [32,33]. By phosphorylating a number of cellular and viral substrates, pUL97 regulates various steps during HCMV replication (reviewed in Marschall *et al.* [3]). In the present study, we refine our previous report on pUL97-cyclin interaction. Specifically, we provide novel data for the high-affinity pUL97-cyclin B1 interaction, with the mode of interaction determined by kinase activity and structural properties of pUL97, and we present the first three-dimensional concept of this previously-unrecognized example of herpesviral kinase-cyclin interaction.

## 2. Materials and Methods

### 2.1. Plasmid Constructs

The construction of expression constructs for pUL97 in wild-type and mutant versions, such as pcDNA-UL97-F, pcDNA-UL97(K355M)-F [30], and pcDNA-UL97(1-595)-F [18], has been described elsewhere. To generate C-terminally truncated versions of pUL97 (1-706, 1-702, 1-692, 1-657), fragments were amplified from the template pcDNA-UL97-F [30]. For amplification primers containing *Xho*I and *Eco*RI restriction sites (synthetic primers purchased from Biomers GmbH, Ulm, Germany) were used and PCR was performed with Vent DNA polymerase (New England Biolabs, Ipswich, MA, USA) under standard conditions [35]. PCR products were subsequently inserted into pcDNA3.1+ (Invitrogen, Carlsbad, CA, USA). A commercially available construct pDsRed1-N1 (BD Clontech, Mountain View, CA, USA) expressing red fluorescent protein was used as a control.

### 2.2. Cell Culture, HCMV Infections and Plasmid Transfection

Human embryonic kidney epithelial cells (293T) were cultivated in Dulbecco’s modified Eagle’s medium containing 10% FCS and primary human foreskin fibroblasts (HFF) in MEM containing 7.5% FCS. HCMV infection experiments were performed at a multiplicity of infection (MOI) of 0.5 to 1.0 using HCMV strain AD169 or AD169-GFP [38]. Transfection of 293T cells was performed using polyethylenimine reagent (Sigma-Aldrich, St. Louis, MO, USA). For the transfection of a 10 cm dish, 10 µg DNA was mixed with 1 mL PBS and subsequently incubated with 20 µL PEI 2000 in 1 mL PBS for 20 min at room temperature. Thereafter, the solution was mixed with 36 µL PEI 25,000 in 1 mL PBS and incubated for 20 min before dropwise addition to the cell layer in a 10 cm dish. After incubation for 4 h at 37 °C, the transfection solution was replaced by fresh medium.

### 2.3. Antibodies

The following polyclonal (pAb) and monoclonal (mAb) antibodies were used: mAb-UL97 (clone 1C4/0.2, produced and kindly provided by Dr. T. Lenac/Prof. S. Jonjic, Dept. Histology and Embryology, Univ. Rijeka, Croatia), pAb-UL97 (06-09, kindly provided by Prof. D. M. Coen, Harvard Medical School, Boston, MA, USA), mAb-UL44 (BS 510, kindly provided by Prof. B. Plachter, Univ. Mainz, Mainz, Germany), mAb-pp65 (65-33, kindly provided by Prof. W. J. Britt, UAB, Birmingham, AL, USA), mAb-β-actin (AC-15, Sigma-Aldrich), mAb-cyclin B1 (sc-7393, Santa Cruz; GNS11, Thermo Fisher Scientific, Waltham, MA, USA), pAb-cyclin B1 (sc-752, Santa Cruz), mAb-cyclin T1 (sc-271348, Santa Cruz), pAb-cyclin T1 (sc-10750, Santa Cruz), pAb-Fc (rabbit Fc fragment, Jackson ImmunoResearch Laboratories, Bar Harbor, ME, USA), mAb-Flag (M2, Sigma-Aldrich), pAb-Flag (F7425, Sigma Aldrich), and mAb-HA (Clone 7, Sigma-Aldrich). The following fluorescent dye-conjugated secondary antibodies were applied in immunofluorescence analyses: Alexa 488-conjugated goat anti-rabbit IgG (H+L) and Alexa 555-conjugated goat anti-mouse IgG (H+L) (Life Technologies, Carlsbad, CA, USA).

### 2.4. Coimmunoprecipitation (CoIP)

The 293T cells were seeded in 10 cm dishes at a cell number of 5 × 10^6^ and were transfected with expression plasmids coding for full-length pUL97 or mutants of pUL97 using the polyethylenimine transfection technique. Red fluorescent protein (RFP; pDsRed1-N1, BD Clontech) was used as a transfection control. HCMV AD169-infected HFFs were cultivated in cell culture flasks and harvested at 4–5 days post-infection, as indicated. Optionally, inhibitors were added to the culture media one day before harvesting. Plasmid-transfected 293T cells and HCMV-infected HFFs were lysed in 500 mL CoIP buffer (50 mM Tris/HCl [pH 8.0], 150 mM NaCl, 5 mM EDTA, 0.5% NP-40, 1 mM PMSF, 2 µg aprotinin mL^−1^, 2 µg leupeptin mL^−1^ and 2 µg pepstatin mL^−1^) for 20–30 min on ice, centrifuged (14,000 rpm, 4 °C, 10–30 min) and incubated for about 3 h at 4 °C under rotation with antibody-coated protein A sepharose beads (100 µL of a 25 mg/mL solution; Sigma-Aldrich) or Dynabeads^®^ Protein A (25 µL per sample; Life Technologies/Novex), respectively. To absorb unspecific protein binding, lysates of 293T cells were incubated for about 1 h at 4 °C with uncoated protein A sepharose beads. The precipitates were washed five times (1 mL each) with CoIP buffer before the samples were subjected to standard Western blot analysis using tag- or protein-specific antibodies for the detection of coimmunoprecipitates and protein expression (ECL staining; New England Biolabs).

### 2.5. Confocal Imaging

HFFs were grown on coverslips and used for infection with HCMV strain AD169. At three days post-infection, cells were fixed with 4% paraformaldehyde solution (10 min, room temperature) and permeabilized by incubation with 0.2% Triton X-100 solution (15 min, 4 °C). Non-specific staining was blocked by incubation with 2 mg/mL human γ-globulin (cohn fraction II, Sigma Aldrich; 40 min, 37 °C). Indirect immunofluorescence staining was performed by stepwise incubation with primary antibodies for 90 min each at 37 °C, followed by incubation with dye-conjugated secondary antibodies (Alexa, Molecular Probes, Inc., Eugene, OR, USA) for 30 min at 37 °C. Cell samples were mounted with Vectashield Mounting Medium containing DAPI and analyzed using a DMI6000 B microscope and a 63× HCX PL APO CS oil immersion objective lens (Leica Microsystems, Wetzlar, Germany). Confocal laser-scanning microscopy was performed with a TCS SP5 microscope (Leica Microsystems). Images were processed using the Meta-Imaging series (Molecular Devices, Sunnyvale, CA, USA) and LAS AF software (version 1.8.2 build 1465; Leica Microsystems).

### 2.6. In Vitro Kinase Assay (IVKA)

*In vitro* protein phosphorylation was investigated by IVKA analyses using immunoprecipitated proteins from lysates of transiently transfected 293T cells (10 cm dish with approx. 5 × 10^6^ cells per reaction) or HCMV-infected HFFs (T175 flask with approx. 5 × 10^6^ cells per reaction). CoIP of protein complexes for combined CoIP-IVKA reactions was performed according to the CoIP procedure described under 2.3 by cell lysis and immunoprecipitation with antibody-coated protein A beads in CoIP buffer. The final washing step was modified compared to normal CoIP, *i.e.*, by washing twice in 500 μL HNTG buffer (50 mM HEPES [pH 7.5], 150 mM NaCl, 1 mM EDTA, 10% glycerin, and 0.1% Triton X-100) and two times in 500 μL kinase basis buffer (20 mM Tris/Cl [pH 7.5], 0.5 mM MnCl_2_). Precipitates were incubated for 30 min at 30 °C in 40 μL kinase assay buffer (basis buffer including 1 μM ATP, 1 mM DTT and 2.5 μCi [γ-^33^P]-ATP, Hartmann Analytic, Braunschweig, Germany) to start *in vitro* protein phosphorylation. Optionally, 1 μL of a purified histone mix H1-4 (Roche, Mannheim, Germany) was added as an exogenous substrate protein. *In vitro* phosphorylation was finally stopped by denaturation at 95 °C for 10 min with 15 μL of 4×-concentrated SDS-PAGE loading buffer. The radiolabeled proteins were subjected to SDS-PAGE and analyzed by autoradiography using a phosphoimager; levels of protein expression and the reliability of specific immunoprecipitation was controlled by Western blot analysis in parallel.

### 2.7. Homology Modeling of pUL97

The crystal structure of human CDK2 (hCDK2) complexed with the quinazoline compound DIN-234325 (PDB code: 2B53) [39] was selected as a template for the homology modeling of pUL97 inhibited by MBV or the quinazoline compound Ax7396. The loop Leu37-Glu40, which was not resolved in PDB entry 2B53, was modeled according to the coordinates of the respective residues from PDB entry 4KD1 [40]. Additionally, two alternative pUL97 models were generated, which were based on the cyclin B-bound conformations of hCDK2 (PDB code: 2JGZ) [41] or hCDK1 (PDB code: 4Y72; [42]). A comparison of the different models will allow an estimate of whether pUL97 may exhibit a similar conformational plasticity as human CDKs. After the sequence alignment of hCDK1, hCDK2 and pUL97, homology models were created with MODELLER 9.14 [43]. Visualization of pUL97 modeling was done with VMD 1.9.1. [44]. Note that a loop formed by the nonconserved amino acids 506-541 of pUL97 is not shown in the model due to a large gap in the sequence alignment.

## 3. Results 

### 3.1. HCMV Protein Kinase pUL97 Shows a Particularly Strong Interaction with Cyclin B1

**Figure 1:**
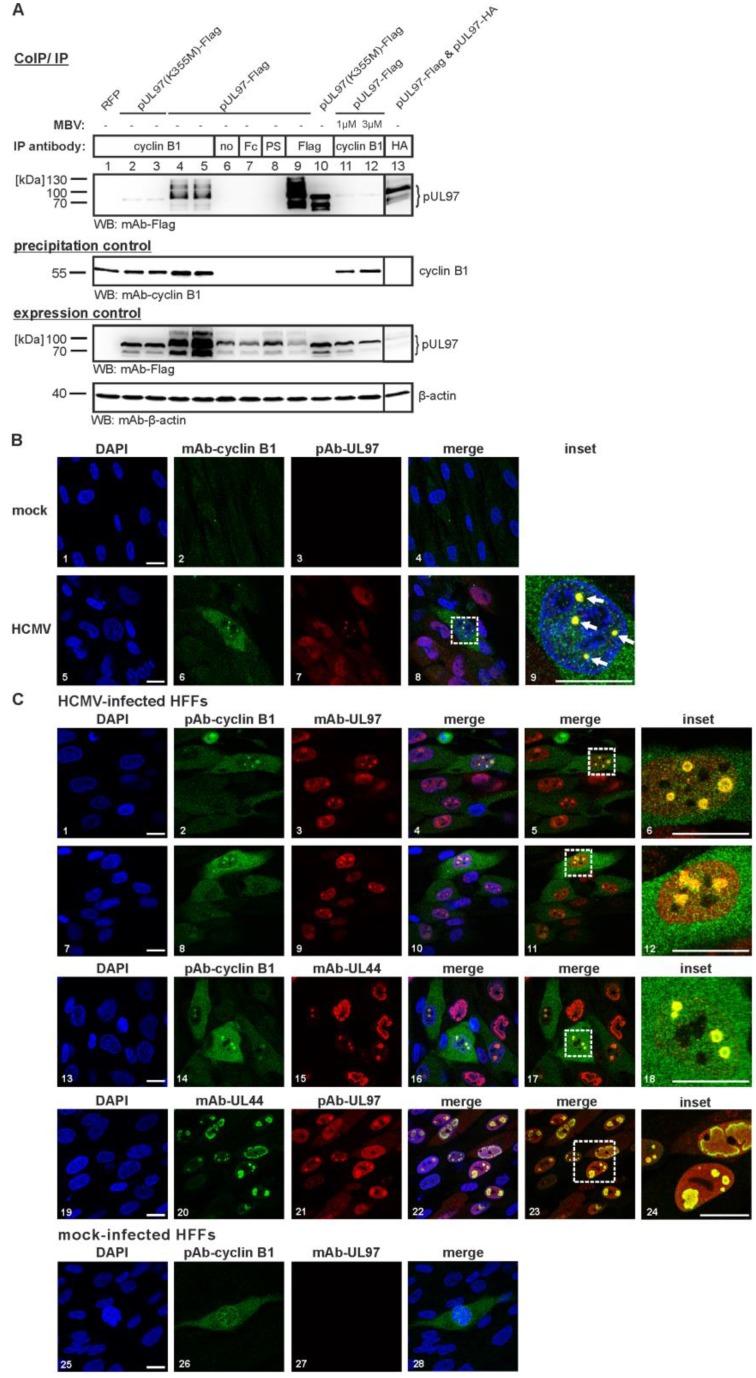
Interaction between HCMV pUL97 and endogenous cyclin B1. (**A**) CoIP analysis showing an activity-dependent interaction between pUL97 and cyclin B1. pUL97-Flag and the catalytically inactive mutant pUL97(K355M)-Flag were transiently expressed in 293T cells. Controls included a separately expressed RFP (transfection control) as well as CoIP specificity controls such as an Fc fragment (Fc), pre-immune serum (PS) and a control sample without antibody (no). Optionally, one day before harvesting the cells MBV was added to the medium (1 µM or 3 µM). At two days post-transfection, cells were lysed and endogenous cyclin B1 was immunoprecipitated using a polyclonal antibody. Coimmunoprecipitates and expression control samples were subjected to Western blot analysis using the antibodies indicated. The precipitation control staining indicated the presence of constant levels of cyclin B1 in the precipitates and the expression control stainings illustrated reliable expression of all relevant proteins. β-actin staining was used as a loading control. CoIP, coimmunoprecipitation; IP antibody, immunoprecipitation antibody; WB, Western blot; (**B**,**C**) Intracellular colocalization between cyclin B1 and pUL97 determined by confocal imaging. HFFs were infected with HCMV AD169 (MOI approx. 0.5, B: panels 5–9, C: panels 1–24) or remained uninfected (B: mock, panels 1–4; fixed 48 h post-infection, hpi). At 72 hpi (**B**) or 96 hpi (**C**), cells were subjected to indirect immunofluorescence analysis using cyclin B1-, pUL44- and pUL97-specific antibodies. Cell nuclei were counterstained with DAPI. Scale bars, 20 µm.

Recently, we identified an interaction of the HCMV-encoded protein kinase pUL97 with cyclin T1, and characterized this interaction with data from yeast two-hybrid, CoIP and kinase assays [45]. In particular, the region responsible for cyclin T1 could be narrowed down to amino acids 231-280 of pUL97. In our ongoing analysis (M.M., unpublished data) and in the previous report by Graf *et al.* [45], we noticed that cyclin interaction of pUL97 was not exclusively restricted to cyclin T1, but also included further cyclin types, such as B1, A, and others, and that these interactions can be detected in HCMV-infected cells. In the present study, we demonstrate an interaction of pUL97 with cyclin B1 that is more prominent than that described for cyclin T1 and that is characterized by several unexpected features. In a CoIP setting, we used a polyclonal anti-cyclin B1 antibody (pAb-cyclin B1) to precipitate endogenous cyclin B1 together with transiently expressed pUL97-Flag (Figure 1A, lane 3). Recently, we reported the formation of up to three isoforms for pUL97 [32,33,34], which are, likewise, nicely detectable in this setting (lanes 9 and 13), whereby the inactive mutant K355M shows an isoform band pattern at reduced molecular masses compared to wild-type due to the lack of autophosphorylation (lane 10). Interestingly, cyclin B1 interaction could also be detected for the isoforms of pUL97 (lanes 4–5; see mainly the abundantly expressed large and middle isoforms M1 and M74; note that, generally, the quantity of detectable levels of pUL97 isoforms, mutants, as well as wild-type is in part a matter of limited protein stability as reported earlier [34]). Specificity of the CoIP setting was illustrated by the use of three negative controls (lanes 6–8; no antibody, Fc fragment, and pre-immune serum, respectively). The pUL97-cyclin B1 interaction was supported by our confocal imaging analysis with HCMV-infected primary fibroblasts. Imaging data demonstrated a colocalization between pUL97 and cyclin B1 in a distinct speckled pattern concentrated in subnuclear compartments (Figure 1B, panels 5–9, compared to a mock control in panels 1–4; Figure 1C, panels 1–12) that were identified as early nuclear viral replication centers using the viral DNA polymerase processivity factor pUL44 as a marker for viral replication centers (panels 13–24). Various combinations of antibodies against pUL97 and cyclin B1 were used to confirm this finding and controls were performed with negative antibodies and mock-infected cells (Figure 1C, panels 25–28; note that in this experiment, at least 50 further mock-infected, cyclin B1-positive cells were detected showing a very similar pattern of cyclin B1 expression). While in mock-infected cells strong cyclin B1 expression was distinctly found in mitotic cells, HCMV-infected cells showed a broad increase in cyclin B1 levels (under the known conditions of HCMV-induced early S phase arrest [11]). A pronounced pUL97-cyclin B1 colocalization was identified in a subnuclear speckled pattern (Figure 1B, panel 9; Figure 1C, panels 6 and 12), also including other viral proteins accumulating in nuclear replication centers, such as pUL44, which is known to interact with and to be phosphorylated by pUL97 ([46]; panels 18 and 24; note that cyclin B1 did not entirely colocalize with pUL44, but that fractions of the two proteins were detected in distinct cells in identical subnuclear compartments). A quantitation of the percentage of cells double-positive for pUL97 and cyclin B1 resulted in 17.8 ± 3.6% of colocalization (when counting exclusively cells with a very prominent signal of colocalization as shown in the inset panels of Figure 1B,C) or, in a total range, 63.2 ± 7.6% (also including cells comprising less prominent colocalization signals; quantitation was achieved by microscopic counting of *n* = 7 of ≥50 cells, mean values ± SD). This finding is in line with previously published data showing pUL97-cyclin T1 colocalization in nuclear viral replication centers [45].

### 3.2. The pUL97-Cyclin B1 Interaction is Dependent on Kinase Activity

**Figure 2:**
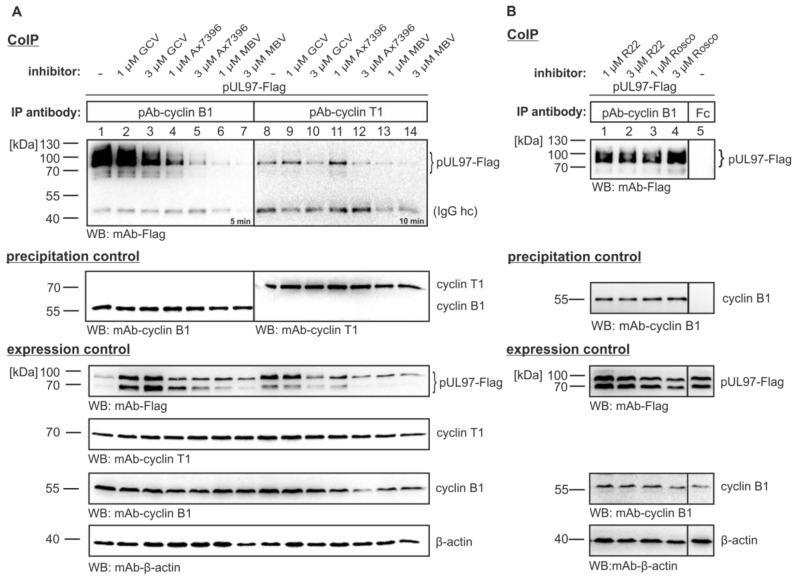
pUL97 inhibitors are able to abrogate interaction with cyclins B1 and T1. (**A**) pUL97-Flag was transiently expressed in 293T cells. Two days post-transfection, reference drug ganciclovir (GCV), pUL97 inhibitors Ax7396 and MBV (**A**) or CDK inhibitors R22 and roscovitine (Rosco) (**B**) were added to the medium at 1 µM and 3 µM. On day three, cells were lysed and endogenous cyclin B1 or cyclin T1 was immunoprecipitated using polyclonal rabbit antibodies. CoIP and expression control samples were subjected to WB analysis using the antibodies indicated (including rabbit antibody Fc fragment as a specificity control). Note that 3 µM of the pUL97-targeting compounds MBV and Ax7396 showed a strong inhibitory effect on cyclin interaction (*i.e.*, the range of anticytomegaloviral activity exerted by these compounds was determined 1–15 µM), so that this concentration was maintained for further experiments.

A striking feature of our current analysis was the finding that inactive pUL97 did not interact with cyclin B1. When CoIP was performed with the catalytically-inactive version pUL97(K355M)-Flag, only a marginal signal was obtained, which stood in contrast to the strong signal of wild-type pUL97 interaction with cyclin B1 (Figure 1A, lanes 2–5). This finding was further supported by the use of pUL97 inhibitors in this CoIP setting (Figure 1A, lanes 11, 12; Figure 2). In the absence of inhibitor, endogenous cyclin B1 interaction was detectable at a particularly high affinity compared to cyclin T1 interaction (Figure 2A, lanes 1 and 8; the specificity of interaction was verified and confirmed by several experiments, using various controls also including pre-immune serum). For both cyclins, interaction could be blocked by treatment with the pUL97 inhibitors Ax7396 [47,48] and MBV [49,50,51]. The block was concentration-dependent and was more prominent for cyclin B1 than for cyclin T1 interaction (Figure 2A, lanes 4–7 and 11–14, respectively). No effect, or only marginal inhibitory effect, on interaction was displayed by the nucleoside analog GCV, which binds as a substrate and is phosphorylated by pUL97 (Figure 2A, lanes 2–3 and 9–10) or by CDK inhibitors R22 [9] and roscovitine [20] (Figure 2B, lanes 1–4). These data point to a strictly activity-determined mode of cyclin interaction, a concept that is particularly supported by the pronounced loss of cyclin B1 interaction in the case of pUL97 mutation K355M and two chemically distinct pUL97-inhibitory small molecules. It is interesting to see that both types of drugs, *i.e.*, the clinical drug candidate MBV and the experimental compound Ax7396 belonging to the quinazoline class, show similar efficacies in blocking pUL97-cyclin B1 interaction. Our recent findings on the novel quinazoline-type inhibitors of pUL97 Vi7392 and Vi7453 (characterized by anti-HCMV 50% effective concentrations in the low micromolar range, EC_50_ ≤ 4 µM; data not shown) indicate an advantageous effect over drugs used in standard therapy, since they appear to be poor inducers of drug resistance. Previously, we reported that three quinazoline compounds did not show an induction of drug resistance against HCMV *in vitro* [47,48]. To address the question of drug resistance for the novel quinazolines Vi7392 and Vi74523, HCMV-infected cells were treated with high drug concentrations (10-fold EC_50_), continuously monitored, and passaged for months. As a striking result, quinazoline-mediated resistance formation was not observed (in contrast to the frequent induction of GCV resistance in control panels). The finding that pUL97 inhibitors of the chemical class quinazolines are non-inducers of pUL97-conferred drug resistance prompted us to hypothesize that quinazolines may differ in their binding mode from other classes of inhibitors. Independent of individual frequencies of drug resistance, these pUL97 inhibitors from different chemical classes had, in common, the ability to block cyclin B1 binding. This suggests that drug binding may be linked with changes in structural conformations of pUL97 as reflected by active and inactive states of the kinase.

**Figure 3:**
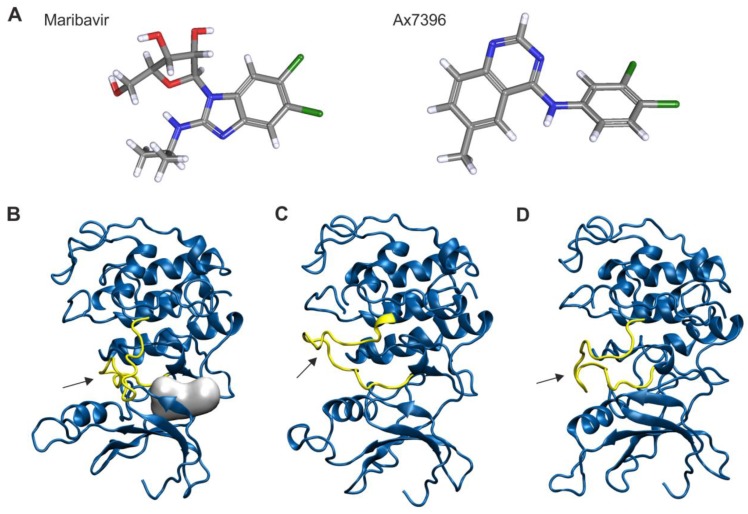
Inhibitors and homology models of the kinase domain of pUL97. (**A**) Structures of MBV and the quinazoline compound Ax7396, which were used as inhibitors; (**B**) Model of the pUL97 kinase. The inhibitor binding site is depicted as grey sphere and the T-loop is shown in yellow; (**C**,**D**) Models of pUL97 kinase that were generated based on cyclin B-bound CDK2 (**C**) or CDK1 (**D**). Comparison to the model of inhibited pUL97 (panel (**B**)) shows that the T-loop (yellow) of pUL97 is sufficiently long that it might adopt alternative conformations (see arrows).

For this purpose, drug-inhibited pUL97 was modeled using the structure of an inhibitor-bound CDK2 as a template. Both MBV and Ax7396 (Figure 3A) fit well into the binding pocket of pUL97 (Figure 3B), supporting the observation that they inhibit pUL97 activity. Therefore, the lack of cyclin binding may be directly related to the absence of autophosphorylation of inhibited pUL97. However, from the basis of crystal structures of human CDKs it is known that inhibitor binding may not only affect the catalytic activity but also the structural properties of CDKs. One example is the conformation of the activation loop, which is rather flexible in CDK1 and CDK2 and constitutes a part of the cyclin B binding interface [42]. In order to explore whether a similar conformational plasticity may also be feasible for pUL97, additional models were generated using the crystal structures of cyclin B-bound CDK1 and CDK2 as templates. The resulting models suggest that the length of the pUL97 activation loop is sufficient to adopt a conformation similar to that observed in CDK1 and CDK2 (Figure 3C,D). Based on this finding, it is tempting to speculate that the pUL97 kinase domain may directly interact with cyclin B in a fashion similar to CDK1 or CDK2. However, further experiments are required to verify this hypothesis.

### 3.3. Serial Deletions of the pUL97 C-Terminus Abrogate Cyclin B1 Interaction

**Figure 4:**
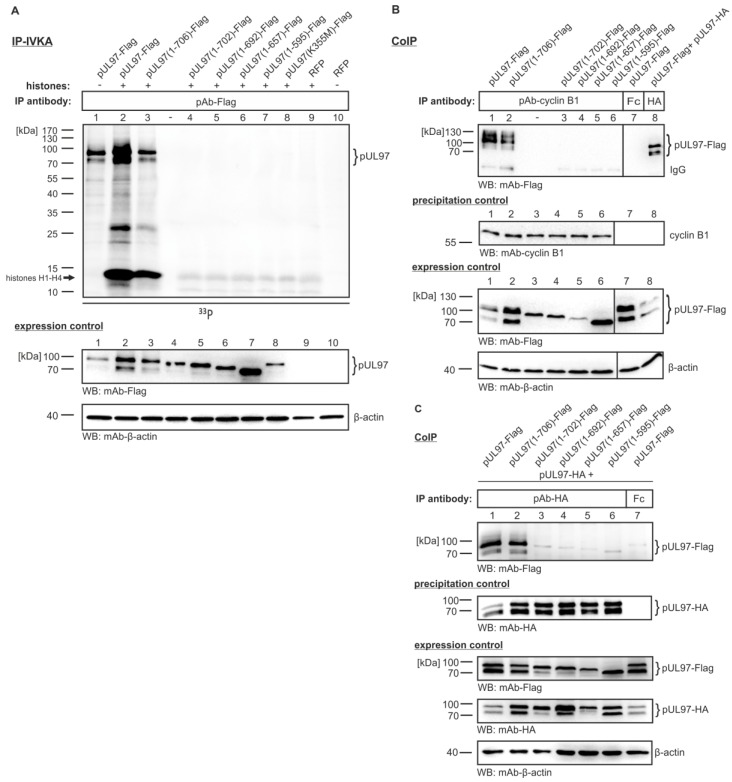
Importance of the C-terminus of pUL97 for kinase activity, cyclin B1 binding and pUL97-pUL97 interaction. Full-length pUL97 and C-terminally truncated versions lacking 1, 5, 15, 50 or 112 amino acids were transiently transfected into 293T cells (concentrations of plasmid DNA were adjusted between 10 µg and 20 µg to achieve homogeneous expression levels). Three days post-transfection, cells were lysed and endogenous cyclin B1 (**B**) or cotransfected pUL97-HA (**C**) was immunoprecipitated. Coimmunoprecipitated proteins (pAb-cyclin B1, positive control mAb-HA, negative controls rabbit Fc fragment and pre-immune serum not shown) were analyzed via WB analysis (**B**,**C**). For measuring *in vitro* kinase activity of C-terminally truncated versions of pUL97, an IVKA was performed with the immunoprecipitates (**A**). In reactions 2–9, a standard substrate of pUL97, *i.e.*, purified histones H1-H4 (comigrating in one band as resulting from the chosen SDS-PAGE conditions), was added exogenously together with [γ-^33^P]-ATP. Phosphorylated proteins were separated by SDS-PAGE/WB and analyzed on a phosphoimager plate.

Our previously published reports implied that an intact C-terminus of pUL97, but not an intact N-terminus, is required for full kinase activity [18,32,34,35]. To prove this suggestion, we generated further C-terminally truncated versions of pUL97 [ORF UL97(1-707) lacking 1, 5, 15, 50 or 112 amino acids] and expressed the proteins in transiently transfected 293T cells. When immunoprecipitated by the use of Flag tag-specific antibodies and analyzed for activity in *in vitro* kinase assays (IP-IVKA), we detected strong autophosphorylation and histone substrate phosphorylation for full length pUL97 (pUL97-Flag; Figure 4A, lanes 1–2) and the construct lacking only one amino acid (pUL97(1-706)-Flag; Figure 4A, lane 3). In contrast, all other constructs carrying further C-terminal truncations completely lacked activity and, thus, were negative in both autophosphorylation and substrate phosphorylation. (Note that the faint bands for histones in lanes 4–8 correlate with background phosphorylation levels shown in lane 9).

Next, we addressed the question whether these catalytically inactive C-terminally truncated mutants of pUL97 were likewise negative for cyclin B1 interaction. In a CoIP setting (similar to the one shown in Figure 2), we performed an immunoprecipitation of endogenous cyclin B1 and searched for those versions of pUL97 that were positive in CoIP. As a striking result, we exclusively obtained positive signals for full-length pUL97(1-707) and pUL97(1-706) (Figure 4B, lanes 1–2), whereas all further truncated versions were negative (lanes 3–6). CoIP controls with an Fc fragment (incapable of recognizing pUL97-Flag) or pAb-HA (indicating self-interaction between pUL97-Flag and pUL97-HA; it should be stressed that this interaction might be indirect, thus possibly bridged by a number of cellular proteins) served to monitor the specificity of reactions (Figure 4B, lanes 7–8). This finding argues for an important role of the C-terminus in pUL97, not only for the folding of an intact catalytic center of the kinase domain but also for cyclin B1 recruitment. Moreover, it was also interesting to see that pUL97-pUL97 interaction (an activity basically mapped to the N-terminal region 231–280; [35]) showed an identical dependence on the intact C-terminus. Here again, those truncations removing more than one amino acid from the C-terminus abrogated pUL97-pUL97 interaction (Figure 4C). Thus, we postulate that the C-terminal region of pUL97, although not contained within the classic kinase domain (defined by conserved subdomains I-XI; [16]), has an overall importance for catalytic activity and protein interactions. Truncations of the C-terminus may lead to structural alterations that are incompatible with normal pUL97 functionality, including phosphorylation activity, cyclin B1 interaction and pUL97-pUL97 interaction. In this regard, it might be relevant that all manipulations described in Figure 1, Figure 2, and Figure 4 (*i.e.*, replacement mutation K355M, treatment with kinase inhibitors and C-terminal truncation, respectively) have one consequence in common, namely to prevent pUL97 autophosphorylation. For this reason, it is tempting to speculate that pUL97 autophosphorylation might be a trigger that induces so far uncharacterized structural changes in order to establish a pUL97 conformation that is competent for cyclin B1 binding.

### 3.4. The Cyclin B1-pUL97 Complex Precipitated from HCMV-Infected Cells Comprises Substrate-Binding and Substrate-Phosphorylating Activity

**Figure 5:**
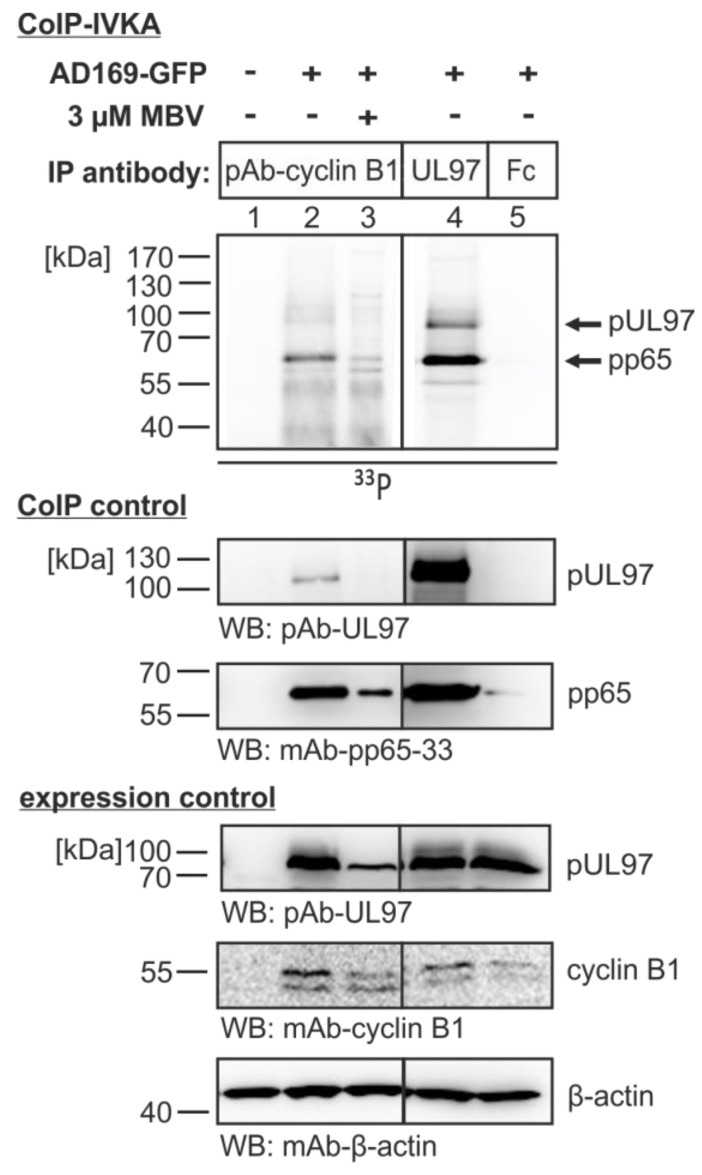
Combined coimmunoprecipitation-*in vitro* kinase assay (CoIP-IVKA) demonstrating the pUL97-specific phosphorylation of proteins in pUL97-cyclin B1 coimmunoprecipitates derived from HCMV-infected cells. HFFs were infected with HCMV AD169-GFP (approx. MOI 1.0). Four days post-infection, 3 µM of the pUL97 inhibitor was added to the medium to address the question of pUL97-specific phosphorylation. On day five, GFP expression was controlled by microscopic inspection before cell lysates were collected. Cyclin B1 (lanes 1–3) or pUL97 (lane 4) was immunoprecipitated using polyclonal antibodies as indicated; nonspecific precipitation was excluded by performing control IPs with Fc fragment (lane 5) and Flag-specific antibodies (not shown). CoIP samples were subjected to an IVKA reaction and phosphorylated proteins were detected by SDS-PAGE/WB and phosphoimager exposure. A restaining of the radioactive blot (CoIP control) and staining of additional expression control WBs indicated the reliability of protein precipitation and expression levels, respectively.

The specific phosphorylation of proteins present in pUL97-cyclin B1 coimmunoprecipitates was investigated by the use of HCMV-infected primary fibroblasts. For this purpose a combined CoIP-IVKA was performed to demonstrate the activity and substrate specificity of pUL97 contained in the cyclin complex (the identity of proteins was confirmed by WB restaining of the identical WB membranes; Figure 5, panels CoIP control). Autophosphorylation of pUL97 and phosphorylation of one of the most abundant viral substrate proteins, pp65 [52], was detected in the control sample derived from direct pUL97 immunoprecipitation (Figure 5, lane 4). Both activities, *i.e.*, auto- and substrate phosphorylation of pUL97, albeit to a lower intensity, were similarly contained in the complex derived from cyclin B1 CoIP (lane 2). An upregulation of distinct CDKs/cyclins during HCMV replication had been described by various reports [11] and this finding was confirmed for cyclin B1 in our experiment, with an increased level in HCMV-infected cells compared to uninfected cells (Figure 5, lanes 2–5 *versus* lane 1, expression control). The pUL97 specificity of the reaction was indicated by suppression with the pUL97-directed inhibitor MBV (lane 3) and the reliability of the setting was monitored by negative controls (lanes 1 and 5). Of note, we did not detect substantial amounts of cyclin B1 phosphorylation by pUL97 (or any cross-phosphorylation by putatively coimmunoprecipitated CDK activity). This lack of detectable amounts of pUL97-specific *in vitro* phosphorylation of cyclin B1 was also confirmed by equivalent settings using other cyclins as CoIP targets (*i.e.*, using antibodies against cyclins T1, A or H; data not shown). This finding was surprising, although it should be mentioned that the IVKA reactions were performed in an assay buffer previously optimized for pUL97 that only allows some residual activity of CDKs or other possibly associated protein kinases. In principle, we favor a model of cyclin-associated higher order protein complexes that might include more than one kinase activity (possibly additional CDK activity), which, however, includes activity regulation of the kinases and possibly changes in substrate specificity (see below, Figure 5). At this point, it appears also possible that pUL97-cyclin interactions within such complexes might at least to some extent be bridged by a third protein. Importantly, the strong phosphorylation signal obtained for pp65 indicated that at least one viral substrate protein is closely associated with the pUL97-cyclin B1 complex in HCMV-infected cells. Further investigation of the nature of such multiprotein complexes is presently being performed by the use of a recently-established, sensitive, tandem mass spectrometry approach [53].

## 4. Discussion

Interactions between herpesviral protein kinases and host cell-derived cyclins have not been well-characterized to date. The first exception was given by the previously identified interaction between HCMV pUL97 and cyclin T1 [45]. This interaction, although not detectable in high abundance but rather at limited levels of signals, was demonstrated for endogenous cyclin T1, both upon pUL97 overexpression and HCMV productive infection. In addition, an interaction with several types of cyclins was also found, *i.e.*, besides cyclin T1 also B1 and A. This finding was confirmed by the data of the present study, stressing the point that pUL97 interaction with cyclin B1 occurs at a level of higher affinity than with cyclin T1. In the initial report by Graf *et al.* [45], the pUL97 region responsible for cyclin T1 interaction was mapped to amino acids 231 to 280, located in the poorly-structured N-terminal portion of pUL97. This cyclin T1-binding region overlaps with the self-interaction domain [35]. The finding already pointed to a possible link between cyclin interaction and pUL97-pUL97 interaction. It was speculated that the formation of pUL97 dimers and oligomers may be bridged through cyclin interaction. This concept has been further substantiated by the important finding of the present report that pUL97-cyclin B1 interaction is strictly dependent on kinase activity. Three points of evidence support this notion, *i.e.*, cyclin interaction was experimentally abrogated by (i) treatment with pUL97 inhibitors; (ii) replacement of an essential lysine of the ATP-binding site (K355M); and (iii) deletion of C-terminal parts of pUL97 also required for catalysis. This indicates that pUL97-cylin interaction is limited by structural properties of an active kinase domain. It is currently unknown whether the binding of a small molecule, such as the applied quinazoline compounds or drug candidate MBV, is able to sterically block the access of the kinase domain to cyclin interaction or whether a block of cyclin interaction is caused by a binding-incompetent conformation of pUL97. However, the finding that three different experimental situations (all associated with an inactive state of pUL97) led to an abrogation of cyclin interaction strongly favors the latter possibility. This is also supported by our bioinformatical investigations providing the first theoretical concept for conformational changes within the pUL97 kinase domain that may directly determine cyclin interaction.

Since our current state of investigations does not suggest a dependency of pUL97 activity on cyclin binding, we postulate a refined model based on the following considerations of the pUL97 structure-activity relation: The biochemical state of *de novo*-synthesized pUL97 might allow for the correct folding of an initial conformation of the kinase domain characterized by a basic level of activity. In a next step, pUL97 might undergo massive autophosphorylation (possibly also including additional transphosphorylation by cellular kinases such as CDKs; reviewed by Marschall *et al.* [3,54]). The state of autophosphorylation is considered a putative trigger for a conformational change that might render the kinase domain accessible to cyclin interaction. This conformational change might also provide full intrinsic activity to the catalytic cleft within the kinase domain. Thus, in a cyclin-associated form, pUL97 might be fully competent for substrate interaction and downstream phosphorylation activity. More biochemical data will be necessary to validate this concept and, in particular, our ongoing research based on mass spectrometry analysis of protein complexes and the detailed investigation of drug-kinase interaction may provide important information in the near future.

## References

[1] Mocarski E.S., Shenk T., Griffiths P.D., Pass R.F., Knipe D.M., Howley P.M. (2013). Cytomegaloviruses. Fields Virology.

[2] Schreiber A., Härter G., Schubert A., Bunjes D., Mertens T., Michel D. (2009). Antiviral treatment of cytomegalovirus infection and resistant strains. Expert Opin. Pharmacother..

[3] Marschall M., Feichtinger S., Milbradt J. (2011). Regulatory roles of protein kinases in cytomegalovirus replication. Adv. Virus Res..

[4] Marschall M., Stamminger T. (2009). Molecular targets for antiviral therapy of cytomegalovirus infections. Future Microbiol..

[5] Prichard M.N. (2009). Function of human cytomegalovirus UL97 kinase in viral infection and its inhibition by maribavir. Rev. Med. Virol..

[6] Chou S. (2008). Cytomegalovirus UL97 mutations in the era of ganciclovir and maribavir. Rev. Med. Virol..

[7] Herget T., Marschall M., Bogner E., Holzenburg A. (2006). Recent developments in anti-herpesviral combination therapy based on protein kinase inhibitors. New Concepts of Antiviral Therapy.

[8] Schang L.M., St. Vincent M.R., Lacasse J.J. (2006). Five years of progress on cyclindependent kinases and other cellular proteins as potential targets for antiviral drugs. Antivir. Chem. Chemother..

[9] Feichtinger S., Stamminger T., Müller R., Graf L., Klebl B., Eickhoff J., Marschall M. (2011). Recruitment of cyclin-dependent kinase 9 to nuclear compartments during cytomegalovirus late replication: Importance of an interaction between viral pUL69 and cyclin T1. J. Gen. Virol..

[10] Lim S., Kaldis P. (2013). Cdks, cyclins and CKIs: Roles beyond cell cycle regulation. Development.

[11] Hertel L., Chou S., Mocarski E.S. (2007). Viral and cell cycle-regulated kinases in cytomegalovirusinduced pseudomitosis and replication. PLoS Pathog..

[12] Hutterer C., Wandinger S.K., Wagner S., Müller R., Stamminger T., Zeitträger I., Godl K., Baumgartner R., Strobl S., Marschall M. (2013). Profiling of the kinome of cytomegalovirus-infected cells reveals the functional importance of host kinases Aurora A, ABL and AMPK. Antivir. Res..

[13] Kapasi A.J., Spector D.H. (2008). Inhibition of the cyclin-dependent kinases at the beginning of human cytomegalovirus infection specifically alters the levels and localization of the RNA polymerase II carboxyl-terminal domain kinases cdk9 and cdk7 at the viral transcriptosome. J. Virol..

[14] Sanchez V., Spector D.H. (2006). Cyclin-dependent kinase activity is required for efficient expression and posttranslational modification of human cytomegalovirus proteins and for production of extracellular particles. J. Virol..

[15] Tamrakar S., Kapasi A.J., Spector D.H. (2005). Human cytomegalovirus infection induces specific hyperphosphorylation of the carboxyl-terminal domain of the large subunit of RNA polymerase II that is associated with changes in the abundance, activity, and localization of cdk9 and cdk7. J. Virol..

[16] Romaker D., Schregel V., Maurer K., Auerochs S., Marzi A., Sticht H., Marschall M. (2006). Analysis of the structure-activity relationship of four herpesviral UL97 subfamily protein kinases reveals partial but not full functional conservation. J. Med. Chem..

[17] Hume A.J., Finkel J.S., Kamil J.P., Coen D.M., Culbertson M.R., Kalejta R.F. (2008). Phosphorylation of retinoblastoma protein by viral protein with cyclin-dependent kinase function. Science.

[18] Marschall M., Marzi A., aus dem Siepen P., Jochmann R., Kalmer M., Auerochs S., Lischka P., Leis M., Stamminger T. (2005). Cellular p32 recruits cytomegalovirus kinase pUL97 to redistribute the nuclear lamina. J. Biol. Chem..

[19] Thomas M., Rechter S., Milbradt J., Auerochs S., Müller R., Stamminger T., Marschall M. (2009). The cytomegaloviral protein kinase pUL97 interacts with the nuclear mRNA export factor pUL69 to modulate its intranuclear localization and activity. J. Gen. Virol..

[20] Rechter S., Scott G.M., Eickhoff J., Zielke K., Auerochs S., Müller R., Stamminger T., Rawlinson W.D., Marschall M. (2009). Cyclin-dependent kinases phosphorylate the cytomegalovirus RNA export protein pUL69 to modulate its nuclear localization and activity. J. Biol. Chem..

[21] Kawaguchi Y., Kato K., Tanaka M., Kanamori M., Nishiyama Y., Yamanashi Y. (2013). Conserved protein kinases encoded by herpesviruses and cellular protein kinase cdc2 target the same phosphorylation site in eukaryotic elongation factor 1delta. J. Virol..

[22] Kawaguchi Y., Matsumura T., Roizman B., Hirai K. (1999). Cellular elongation factor 1δ is modified in cells infected with representative alpha-, beta-, or gammaherpesviruses. J. Virol..

[23] Milbradt J., Webel R., Auerochs S., Sticht H., Marschall M. (2010). Novel mode of phosphorylation-triggered reorganization of the nuclear lamina during nuclear egress of human cytomegalovirus. J. Biol. Chem..

[24] Milbradt J., Auerochs S., Sticht H., Marschall M. (2009). Cytomegaloviral proteins that associate with the nuclear lamina: Components of a postulated nuclear egress complex. J. Gen. Virol..

[25] Hamirally S., Kamil J.P., Ndassa-Colday Y.M., Lin A.J., Jahng W.J., Baek M.C., Noton S., Silva L.A., Simpson-Holley M., Knipe D.M. (2009). Viral mimicry of Cdc2/cyclin-dependent kinase mediates disruption of nuclear lamina during human cytomegalovirus nuclear egress. PLoS. Pathog..

[26] Prichard M.N., Sztul E., Daily S.L., Perry A.L., Frederick S.L., Gill R.B., Hartline C.B., Streblow D.N., Varnum S.M., Smith R.D. (2008). Human cytomegalovirus UL97 kinase activity is required for the hyperphosphorylation of retinoblastoma protein and inhibits the formation of nuclear aggresomes. J. Virol..

[27] Baek M.C., Krosky P.M., Pearson A., Coen D.M. (2004). Phosphorylation of the RNA polymerase II carboxyl-terminal domain in human cytomegalovirus-infected cells and *in vitro* by the viral UL97 protein kinase. Virology.

[28] Marschall M., Stein-Gerlach M., Freitag M., Kupfer R., van den Bogaard M., Stamminger T. (2002). Direct targeting of human cytomegalovirus protein kinase pUL97 by kinase inhibitors is a novel principle of antiviral therapy. J. Gen. Virol..

[29] Prichard M.N., Gao N., Jairath S., Mulamba G., Krosky P., Coen D.M., Parker B.O., Pari G.S. (1999). A recombinant human cytomegalovirus with a large deletion in UL97 has a severe replication deficiency. J. Virol..

[30] Marschall M., Stein-Gerlach M., Freitag M., Kupfer R., van den Bogaard M., Stamminger T. (2001). Inhibitors of human cytomegalovirus replication drastically reduce the activity of the viral protein kinase pUL97. J. Gen. Virol..

[31] Hanks S.K., Quinn A.M., Hunter T. (1988). The protein kinase family: Conserved features and deduced phylogeny of the catalytic domains. Science.

[32] Webel R., Milbradt J., Auerochs S., Schregel V., Held C., Nöbauer K., Razzazi-Fazeli E., Jardin C., Wittenberg T., Sticht H., Marschall M. (2011). Two isoforms of the protein kinase pUL97 of human cytomegalovirus are differentially regulated in their nuclear translocation. J. Gen. Virol..

[33] Webel R., Solbak S.M.Ø., Held C., Milbradt J., Groß A., Eichler J., Wittenberg T., Jardin C., Sticht H., Fossen T. (2012). The nuclear import of isoforms of the cytomegalovirus kinase pUL97 is mediated by differential activity of NLS1 and NLS2 both acting through classical importin-α binding. J. Gen. Virol..

[34] Webel R., Hakki M., Prichard M.N., Rawlinson W.D., Marschall M., Chou S. (2014). Differential properties of cytomegalovirus pUL97 kinase isoforms affect viral replication and maribavir susceptibility. J. Virol..

[35] Schregel V., Auerochs S., Jochmann R., Maurer K., Stamminger T., Marschall M. (2007). Mapping of a self-interaction domain of the cytomegalovirus protein kinase pUL97. J. Virol..

[36] Baek M.C., Krosky P.M., Coen D.M. (2002). Relationship between Autophosphorylation and Phosphorylation of Exogenous Substrates by the Human Cytomegalovirus UL97 Protein Kinase. J. Virol..

[37] He Z., He Y.S., Kim Y., Chu L., Ohmstede C., Biron K.K., Coen D.M. (1997). The human cytomegalovirus UL97 protein is a protein kinase that autophosphorylates on serines and threonines. J. Virol..

[38] Marschall M., Freitag M., Weiler S., Sorg G., Stamminger T. (2000). Recombinant green fluorescent protein-expressing human cytomegalovirus as a tool for screening antiviral agents. Antimicrob. Agents Chemother..

[39] Sielecki T.M., Johnson T.L., Liu J., Muckelbauer J.K., Grafstrom R.H., Cox S., Boylan J., Burton C.R., Chen H., Smallwood A. (2001). Quinazolines as cyclin dependent kinase inhibitors. Bioorg. Med. Chem. Lett..

[40] Martin M.P., Olesen S.H., Georg G.I., Schonbrunn E. (2013). Cyclin-dependent kinase inhibitor dinaciclib interacts with the acetyl-lysine recognition site of bromodomains. ACS Chem. Biol..

[41] Brown N.R., Lowe E.D., Petri E., Skamnaki V., Antrobus R., Johnson L.N. (2007). Cyclin B and cyclin A confer different substrate recognition properties on CDK2. Cell Cycle.

[42] Brown N.R., Korolchuk S., Martin M.P., Stanley W.A., Moukhametzianov R., Noble M.E.M., Endicott J.A. (2015). CDK1 structures reveal conserved and unique features of the essential cell cycle CDK. Nat. Commun..

[43] Webb B., Sali A. (2014). Comparative Protein Structure Modeling Using MODELLER. Curr. Protoc. Bioinform..

[44] Humphrey W., Dalke A., Schulten K. (1996). VMD: Visual molecular dynamics. J. Mol. Graph..

[45] Graf L., Webel R., Wagner S., Hamilton S.T., Rawlinson W.D., Sticht H., Marschall M. (2013). The cyclin-dependent kinase ortholog pUL97 of human cytomegalovirus interacts with cyclins. Viruses.

[46] Marschall M., Freitag M., Suchy P., Romaker D., Kupfer R., Hanke M., Stamminger T. (2003). The protein kinase pUL97 of human cytomegalovirus interacts with and phosphorylates the DNA polymerase processivity factor pUL44. Virology.

[47] Herget T., Freitag M., Morbitzer M., Kupfer R., Stamminger T., Marschall M. (2004). Novel chemical class of pUL97 protein kinase-specific inhibitors with strong anticytomegaloviral activity. Antimicrob. Agents Chemother..

[48] Schleiss M., Eickhoff J., Auerochs S., Leis M., Abele S., Rechter S., Choi Y., Anderson J., Scott G., Rawlinson W. (2008). Protein kinase inhibitors of the quinazoline class exert anticytomegaloviral activity *in vitro* and *in vivo*. Antivir. Res..

[49] Biron K.K., Harvey R.J., Chamberlain S.C., Good S.S., Smith A.A., Davis M.G., Talarico C.L., Miller W.H., Ferris R., Dornsife R.E. (2002). Potent and selective inhibition of human cytomegalovirus replication by 1263W94, a benzimidazole L-riboside with a unique mode of action. Antimicrob. Agents Chemother..

[50] Price N.B., Prichard M.N. (2011). Progress in the development of new therapies for herpesvirus infections. Curr. Opin. Virol..

[51] Chou S. (2008). Cytomegalovirus UL97 mutations in the era of ganciclovir and maribavir. Rev. Med. Virol..

[52] Becke S., Fabre-Mersseman V., Aue S., Auerochs S., Sedmak T., Wolfrum U., Strand D., Marschall M., Plachter B., Reyda S. (2010). Modification of the major tegument protein pp 65 of human cytomegalovirus inhibits viral growth and leads to the enhancement of a protein complex with pUL69 and pUL97 in infected cells. J. Gen. Virol..

[53] Milbradt J., Kraut A., Hutterer C., Sonntag E., Schmeiser C., Ferro M., Wagner S., Lenac T., Claus C., Pinkert S. (2014). Proteomic analysis of the multimeric nuclear egress complex of human cytomegalovirus. Mol. Cell Proteomics.

[54] Hutterer C., Eickhoff J., Milbradt J., Korn K., Zeitträger I., Bahsi H., Wagner S., Zischinsky G., Wolf A., Degenhart C. (2015). A novel CDK7 inhibitor of the pyrazolo-triazine class exerts broad-spectrum antiviral activity at nanomolar concentrations. Antimicrob. Agents Chemother..

